# Supplementary material for: The Association between Malnutrition and Oral Health in Older People: A Systematic Review
Source: Nutrients. 2021 Oct 13;13(10):3584. doi: 10.3390/nu13103584 (PMC8541038; doi:10.3390/nu13103584)
Supplement: Supplementary file 1 [file nutrients-13-03584-s001.zip › nutrients-1365012-supplementary.pdf]

The association between malnutrition and oral health in older people: A systematic review

Supplementary Table S1. Methodological quality assessment of the included studies

First author	Representative-ness of sample	Sample size	Ascertainment of exposure	Non- respondents	Comparability/ controlling for confounding	Assessment of outcome	Statistical test	Overall quality score
Andersson	*	*	**	*	*	**	*	9
Bøhmer [1]	*	*	**	-	-	**	-	6
El Osta	*	*	**	*	**	*	*	9
Huppertz	*	*	**	*	**	*	*	9
Kiesswetter	*	*	*	*	**	*	*	8
Lindmark	-	*	**	*	*	**	*	8
Mesas	*	*	**	*	**	**	*	10
Pavlović [2]	*	*	*	-	-	**	-	5
Poisson	*	*	**	-	**	**	*	9
Samnieng	*	*	**	-	**	**	*	9
Soini	*	*	**	*	*	**	*	9
Takahashi	*	*	**	-	**	**	*	9

[1,2] excluded for further analyses due to moderate methodological quality score
